# A Cross-Sectional Seroepidemiological Study on Varicella Zoster Virus in Zhejiang, China

**DOI:** 10.3390/vaccines14080722

**Published:** 2026-08-21

**Authors:** Yingying Yang, Yan Liu, Canjie Zheng, Yao Zhu, Fuxing Chen, Linling Ding, Yu Hu, Xiaohua Qi, Yang Zhou

**Affiliations:** 1 Department of Immunization Program, Hangzhou Center for Disease Control and Prevention (Hangzhou Health Supervision Institution), Hangzhou 310021, China; yangyy@hzcdc.com.cn (Y.Y.); liuyan@hzcdc.com.cn (Y.L.); 2 Department of Immunization Program, Quzhou Center for Disease Control and Prevention (Quzhou Health Supervision Institution), Quzhou 324000, China; yangyingying76@163.com; 3 Department of Immunization Program, Zhejiang Provincial Center for Disease Control and Prevention, Hangzhou 310051, China; yzhu@cdc.zj.cn (Y.Z.); fxchen@cdc.zj.cn (F.C.); llding@cdc.zj.cn (L.D.); yhu@cdc.zj.cn (Y.H.); xhqi@cdc.zj.cn (X.Q.)

**Keywords:** varicella, varicella zoster virus, seroprevalence, vaccination, antibody

## Abstract

**Objectives**: This study aimed to investigate the levels of varicella antibodies in Zhejiang, China, after a decade of implementing the two-dose varicella vaccine (VarV) strategy. **Methods**: Healthy participants aged 1 to 69 years from Zhejiang, China, were recruited from January to April 2024 using multistage stratified random sampling, and varicella zoster virus (VZV) IgG antibodies were detected by ELISA. We assessed the seroprevalence and geometric mean concentration (GMC) of VZV IgG and analyzed related factors using Logistic and linear regression models. **Results**: A total of 807 participants were included in the study. The overall seropositivity rate for VZV IgG in the healthy population was 73.08% (95%CI: 69.87–76.11), with a corresponding GMC of 200.97 mIU/mL (95%CI: 182.75 mIU/mL–221.01 mIU/mL). Multivariate analysis identified age group, VarV immunization history, and history of varicella disease as factors significantly associated with both VZV IgG seropositivity and GMC levels (*p* < 0.05). The seropositivity rate for VZV IgG in children aged 1–14 years was 42.67% (95%CI: 36.22–49.31), with a corresponding GMC of 82.10 mIU/mL (95%CI: 69.14 mIU/mL–97.48 mIU/mL). Furthermore, a significant decline in both seropositivity and GMC was observed over time following vaccination among vaccinated children aged 1–14 years (*p* < 0.05). **Conclusions**: Two-dose VarV vaccination may be considered for inclusion in the national immunization program.

## 1. Introduction

Varicella is an acute and highly contagious disease caused by varicella zoster virus (VZV) [1]. VZV is mainly transmitted through droplets, aerosols, or direct contact with rashes and respiratory secretions. VZV primarily targets the susceptible population of children below 14 years of age, giving rise to outbreaks in schools and childcare institutions [2]. Although most varicella symptoms are self-limiting, severe cases can lead to life-threatening complications such as pneumonia, encephalitis, and hemorrhagic conditions [3].

The disease burden caused by varicella is severe, with an estimated 140 million cases occurring annually worldwide, including 4.2 million severe cases requiring hospitalization and 4200 deaths [4]. In China, the disease burden of varicella has risen markedly, exemplified by a surge in annual incidence from 3/100,000 to 70/100,000 between 2005 and 2015 [5]. In Zhejiang, overall varicella incidence dropped from 82.80/100,000 in 2019 to 47.35/100,000 in 2022, while the proportion of cases aged >15 years increased annually [6].

Vaccination with the varicella attenuated live vaccine (VarV) is the most cost-effective public health measure to prevent varicella [7]. VarV is now part of national immunization programs in many countries worldwide [8]. However, VarV is not included in the National Immunization Programme in most regions of China, meaning it is voluntary and self-funded [9]. The estimated VarV coverage in China is 61%, which remains significantly below the World Health Organization (WHO)-recommended target of 80% [10].

Most of serosurveillance studies for VZV infection come from children and adolescents; reports from the general population are rare. Seropositivity and geometric mean concentrations (GMC) were 67.84% and 190.97 mIU/mL, respectively, in healthy children receiving two doses of VarV in Wuxi, China [11]. Among healthy individuals aged 3 to 20 years, the overall seropositivity rate for VZV immunoglobulin G (IgG) was 57.16%, with a median concentration of 136.46 mIU/mL in Quzhou, China [12].

To investigate VZV IgG levels and identify immunologically vulnerable groups in the healthy population aged 1 to 69 years in Zhejiang, 10 years after the implementation of a two-dose VarV strategy, we conducted a serosurveillance study from January to April 2024. The findings provide a scientific basis for optimizing national immunization strategies for VZV.

## 2. Materials and Methods

### 2.1. Study Design

From January to April 2024, we conducted a study, with a cross-sectional design, in Zhejiang. Zhejiang is an eastern–coastal province of China, with an area of 105,500 km^2^ and a population of approximately 66.7 million people. A stratified cluster random sampling method was used to select two prefecture-level cities (Hangzhou and Quzhou) based on geographic location and population composition. We then randomly selected two counties in each city. For children aged 1–14 years who had received VarV, we divided them into five groups according to the time interval after the latest VarV: <1 year, 1-year, 2-year, 3-year, and 4-year. The Ethics Committee of the Chinese Center for Disease Control and Prevention reviewed and approved this protocol (approval code: 202337; approval date: 28 December 2023). Informed consent was obtained from all participants.

### 2.2. Vaccine

Since a live attenuated varicella vaccine became available in Zhejiang Province in 1998, it has been gradually incorporated into its local routine vaccination programs. At 12 months of age, a single dose of VarV is advised. The two-dose vaccination policy, which was put into effect in 2014, advised that children receive the first dose of VarV between the ages of 12 and 18 months and the second dose between the ages of 3 and 4 years, with a minimum of 3 months between doses. During the study period, the overall varicella vaccination coverage rate among the eligible population in Zhejiang Province was 56.27%.

### 2.3. Sample Size Estimation

Based on the sample size formula for the status quo survey [13],

(1) $$N=\frac{p(1\;-\;p)}{({\frac{d}{Z_{\alpha }})}^{2}}$$

where *p* = 0.7, d = 0.1, *p*, α = 0.05, and Z_α_ = 1.96, and taking into account a certain dropout rate, the sample size was increased by 20%, resulting in a total of 200 participants at each survey site. A total of 800 participants were required for all 4 survey sites.

The participants were stratified according to the age ranges of 1–6 years old, 7–12 years old, 13–17 years old, 18–29 years old, 30–39 years old, 40–49 years old, 50–59 years old, and 60–69 years old. At each survey site, 25 participants were enrolled for each age group.

### 2.4. Inclusion and Exclusion Criteria

The inclusion criteria included the following: (1) aged 1 to 69 years with informed consent for blood collection, (2) local residents for at least 3 months, and (3) in good physical health.

The exclusion criteria included the following: (1) refused collection of venous blood and (2) those suffering from acute diseases, severe chronic diseases, acute exacerbation of chronic diseases, immunocompromised diseases, and fever.

### 2.5. Information Collection

A questionnaire survey was used to gather the participants’ basic information, which included their name, gender, age, residence, and history of varicella disease. The questionnaire was completed by the participants’ guardians if they were minors. Vaccination information of the electronic records was obtained from the Zhejiang Provincial Immunization Information System established in 2005. The demographic data were obtained from the Statistical Yearbook published by the Zhejiang Provincial Bureau of Statistics.

### 2.6. Serum Sampling

Three milliliters of peripheral venous blood were drawn from the participants. Initially, all blood samples were centrifuged for five minutes at 3000 rpm. The serum was moved to a new sterilizing tube that was labeled with the blood collection date and sample name. Prior to testing, all serum samples were frozen at −20 °C.

### 2.7. Varicella Zoster Virus IgG Antibody (Anti-VZV IgG) Test

All samples were subjected to enzyme-linked immunosorbent assay (ELISA) using the SERION ELISA classic Varicella zoster virus IgG antibody detection kit (Virion/Serion, Wurzburg, Germany) to detect serum VZV IgG antibodies. Strict adherence to the kits’ manual instructions was maintained throughout the testing process. The positive control was the standard serum containing anti-VZV IgG in PBS, while the negative control was PBS devoid of anti-VZV IgG. After that, each well received 100 µL of IgG enzyme-labeled antibodies and incubated for 30 min at 37 °C. Following one-cycle washing, each well received 100 µL of each substrate, followed by incubation once more for 30 min at 37 °C. Lastly, termination solution (100 µL) was added. All the ELISA results are provided as the OD (optical density) value at the wavelength λ = 405 nm. Using SERION software, the antibody concentration values (mIU/mL) were calculated from the OD value with anti-VZV IgG-positive. The quantitative detection range of VZV-IgG is 15–2000 mIU/mL, with VZV-IgG > 100 mIU/mL being classified as positive and <50 mIU/mL as negative, while 50–100 mIU/mL is equivocal. Retests were conducted on equivocal samples whose concentrations fell between low-high cutoff value; those whose results remained in the gray area were ultimately classified as negative.

### 2.8. Statistical Analysis

The differences of antibody seropositivity within groups were compared using Pearson’s chi-square test. A two independent variable *t*-test or one-way analysis of variance was used to assess the statistical significance of antibody concentrations between groups. Logistic regression was used to analyze factors associated with VZV IgG antibody seropositivity, while multiple linear regression was used to analyze factors associated with GMC. Statistical analyses were performed using R version 4.3.2, and *p* < 0.05 indicated the statistical significance.

## 3. Results

### 3.1. Basic Information

A total of 807 participants aged 1 to 69 years were enrolled in the study. In terms of sex, there were 381 (47.21%) males and 426 (52.79%) females, and the urban-to-rural population ratio was 0.58:1 (295:512). In terms of VarV history, 82 (10.16%) participants received one dose of vaccine and 141 (17.47%) participants received two doses of vaccine; 76 (9.42%) participants in this study reported having varicella.

### 3.2. Factors Associated with Seroprevalence and GMC of VZV IgG

The VZV IgG seropositivity rate was 73.08% (95%CI: 69.87–76.11), and the GMC was 200.97 mIU/mL (95%CI: 182.75 mIU/mL–221.01 mIU/mL). Univariate analysis revealed that several factors were associated with a significantly higher positive rate of varicella antibodies, including old age, lack of vaccination, and history of varicella disease. After adjusting for potential confounders, old age (60–69 years vs. 1–6 years, OR: 55.43, 95%CI: 15.28–201.06), two doses of VarV vaccination (2 dose vs. no, OR: 1.99, 95%CI: 1.11–3.56), and a history of varicella disease (OR: 3.58, 95%CI: 1.43–8.94) were all associated with higher VZV IgG seropositivity (Table 1). 

The differences in the GMC of VZV IgG between age, history of varicella immunization, and history of varicella disease showed similar results. After adjusting for potential confounders, old age (60–69 years vs. 1–6 years, β: 1.82, 95%CI: 1.42–2.23), two doses of VarV vaccination (2 dose vs. no, β: 0.62, 95%CI: 0.29–0.95), and a history of varicella disease (β: 0.43, 95%CI: 0.14–0.73) were all associated with higher GMC (Table 2).

### 3.3. Vaccination Status and VZV IgG Response in Children Aged 1–14 Years

**Figure 1:**
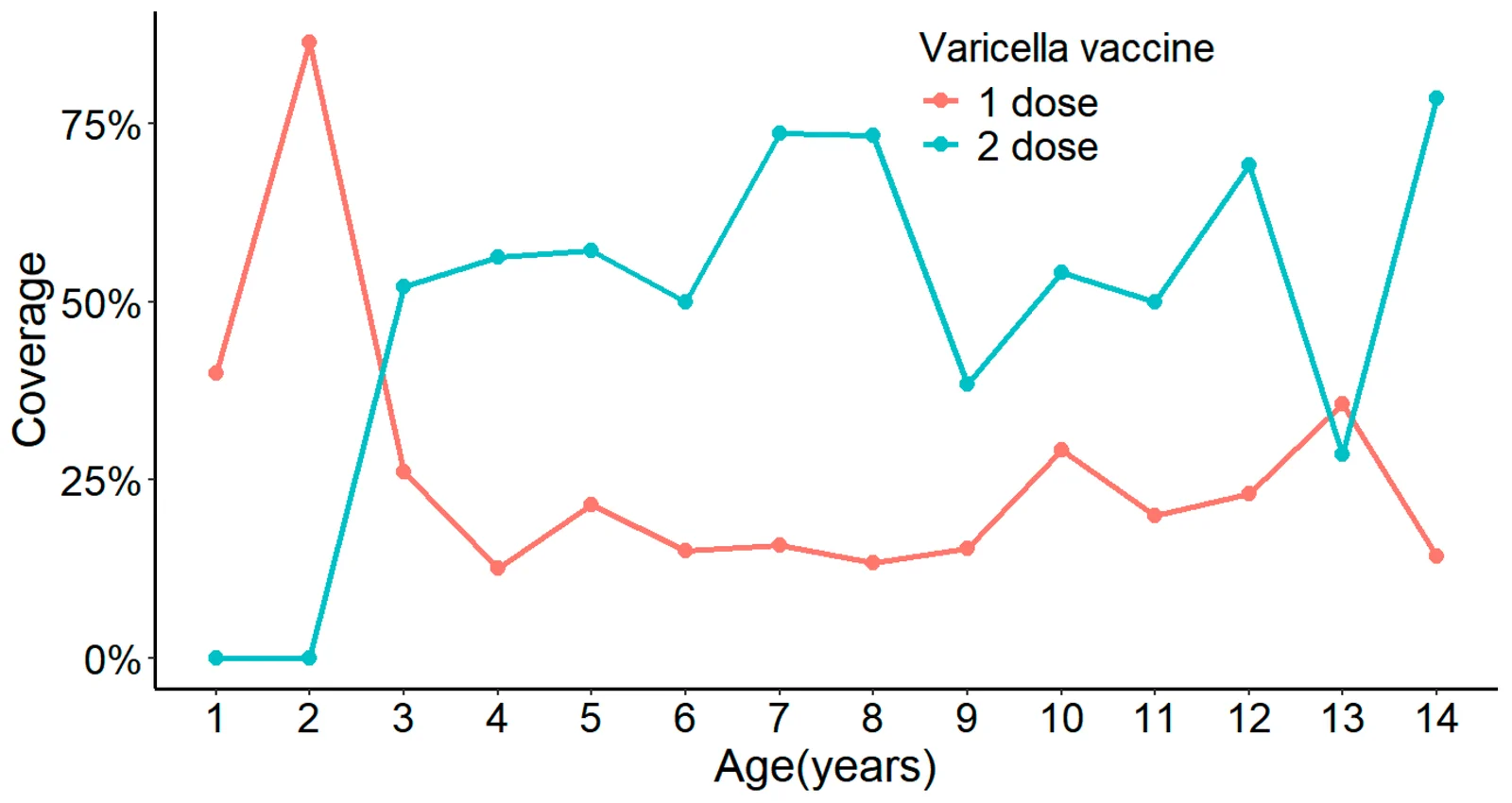
The coverage of the varicella vaccine for participants aged 1–14 years.

In this study, 232 children aged 1–14 years were included, with VarV vaccination rates of 27.16% for one dose and 50.00% for two doses. Among children aged 1–2 years, the one-dose VarV vaccination rate was 77.78%, while 53.85% of children aged 3–4 years received two doses of VarV (Figure 1). The seropositivity rate for VZV IgG in children aged 1–14 years was 42.67% (95%CI: 36.22–49.31), with a corresponding GMC of 82.10 mIU/mL (95%CI: 69.14 mIU/mL–97.48 mIU/mL). The GMC of VZV IgG increased with the doses of VarV (F = 20.32, *p* < 0.001). Additionally, compared with the unvaccinated group, the two-dose group had a significantly higher VZV IgG seropositivity rate (χ^2^ = 8.240, *p* = 0.016) (Table 3).

### 3.4. VZV IgG Seropositivity and GMC Across Different Post-Vaccination Intervals

According to the different time intervals between sampling and vaccination, 179 of the 232 children aged 1–14 years who had received VarV (with a mean vaccination age of 1.49 ± 0.85 years) were divided into five groups: <1 y, 1-y, 2-y, 3-y, and 4-y. The VZV IgG seropositivity in vaccinated participants was 46.37% (95%CI: 38.90–53.96%), and the GMC was 95.10 mIU/mL (95%CI: 78.71 mIU/mL–114.90 mIU/mL). After 4 years following vaccination, the VZV IgG seropositivity dropped to 36.07% (95%CI: 24.16–49.37%), indicating that seropositivity tended to decline over time (*p* = 0.008). Similarly, the GMC was reduced to 71.10 mIU/mL (95%CI: 50.11 mIU/mL–100.87 mIU/mL) after 4 years since vaccination, suggesting that GMC had a tendency to wane with time (Table 4).

## 4. Discussion

Given that the incidence of chickenpox has been rising and varicella outbreaks are increasingly reported worldwide, this has emerged as a significant public health concern [14,15,16]. To address this issue, a cross-sectional serosurveillance study was conducted to assess varicella antibody levels among healthy individuals aged 1 to 69 years in Zhejiang. Monitoring in VZV IgG antibody levels after the implementation of vaccination programs is highly important, as mathematical models of disease transmission have demonstrated that gradual accumulation of susceptible subjects could lead to resurgence of disease after many years of low incidence [17,18]. Therefore, this survey is essential for understanding the current immune status of the population and for informing the development of more targeted varicella vaccination strategies. The findings will provide a scientific basis for refining public health policies and improving vaccination coverage in the region.

The VZV antibody is a crucial marker for assessing the immunization impact following vaccination or infection. The results of this study showed that the total positivity rate of anti-VZV IgG (73.08%) in the healthy population aged 1 to 69 years in Zhejiang was higher than that in another previous study in Chongqing in subjects aged 0–89 years (61.3%) [19]. Meanwhile, the GMC in the present study was also higher than that in Chongqing (200.97 mIU/mL vs. 125.2 mIU/mL). Domestic varicella vaccines were introduced in 1998, a two-dose schedule has been suggested in various parts of China since 2012 [20]. As the public awareness and acceptance of the VarV have increased, the vaccination rate of the VarV in China has generally improved [21,22]. We found that the VarV vaccination rate in participants aged 1–14 years was 77.16%, which was higher than those in Wuxi (67.43%) [11] and Hangzhou (70.06%) [23]. The different coverage of VarV, different sample survey objects and periods, and incidence of varicella may explain the inconsistency in study findings.

Studies have shown that the seroprevalence of VZV IgG antibodies and GMC increased with age. We found that the antibody positivity rate and GMC were lowest in the 1–6 years age group (41.00%, 74.05 mIU/mL) and highest in the 60–69 group (96.91%, 378.98 mIU/mL). Both the seroprevalence of VZV IgG antibodies and GMC levels exhibited an increasing trend with age, potentially due to increased social activities and a higher likelihood of contracting varicella or receiving vaccination as age advances, leading to elevated antibody positivity rates and GMC levels. However, protection against VZV relies largely on cell-mediated immunity rather than humoral antibody levels alone; accordingly, serological measures have inherent limitations in predicting breakthrough disease, as breakthrough varicella can occur even in the presence of detectable IgG. In addition, with the increasing coverage of varicella vaccination, the incidence of varicella among children has gradually decreased, while the proportion of adult cases has increased [24,25,26]. The lower VZV IgG antibody positivity rate and GMC level in the 1–6 years age group compared to older age groups are consistent with findings from related studies, indicating that antibody levels in children within this age group are insufficient to provide protective effects [27,28]. Therefore, high coverage of varicella vaccination should be maintained, as an outbreak of varicella would inevitably affect older age groups, resulting in a more severe disease burden [29].

In the univariate analysis, unvaccinated individuals showed higher seropositivity (83.56%) and GMC (269.92 mIU/mL) than one-dose (34.15%, 60.39 mIU/mL) and two-dose vaccinated groups (51.77%, 119.25 mIU/mL). This discrepancy is most likely explained by a higher proportion of prior natural infection in the unvaccinated group, which typically induces higher antibody levels than vaccine-induced responses. Notably, multivariate analysis revealed significantly higher seropositivity and GMC in the two-dose vaccinated group compared to the unvaccinated group. However, no statistically significant differences were observed between the one-dose vaccinated and unvaccinated groups. Seropositivity was approximately two times higher among two-dose recipients, consistent with findings from other studies [11,12]. Xu et al. [23] reported a 98.89% seroconversion rate with four-fold higher antibody levels after the second dose. These findings support the idea that two-dose VarV vaccination enhances immune response, ensures long-term protection, and is preferable for achieving sufficient population immunity to prevent varicella outbreaks.

Similarly, VZV IgG antibody seroprevalence and GMC levels were higher among individuals with a history of varicella [19,30]. Compared with those without a history of varicella, seropositivity was significantly higher in individuals with prior infection (OR: 3.58, 95%CI: 1.43–8.94). This difference confirms the role of natural infection in inducing high-titer antibodies and underscores the need for high vaccination coverage in uninfected populations to establish an effective immune barrier and reduce the risk of future outbreaks.

Studies have shown that the VZV IgG seropositivity increased with the doses of VarV [11,31]. We found that the rate of VZV IgG seropositivity in children aged 1–14 years was 30.19%, 36.51%, and 51.72% for zero-dose, single-dose, and two-dose applications of VarV. An observational study in Wuxi, China, involving children aged <7 years reported that the antibody-positive rate was highest in subjects who had been immunized with two doses of VarV (67.84%) compared to those with one dose or no inoculation [11]. Ng et al. [31] reported an overall seropositivity rate of 52.9% among participants aged 1–17 years in Singapore, with positivity rates after one and two doses of varicella vaccine of 51.2% and 73.1%, respectively. The GMC in children aged 1–14 years in the present study (82.10 mIU/mL) was lower than that in Quzhou (134.44 mIU/mL) [12] and Harbin (447 mIU/mL) [32]. A number of important factors, such as differences in vaccination rates, time since immunization, laboratory testing methods or kit types, and distinct demographic and regional distributions, may be linked to the comparatively lower levels of seropositivity rate and GMC.

In addition, our study suggested a decline in both VZV IgG seropositivity and GMC over time following vaccination, consistent with the results reported by Wang et al. [11] in 2024 and Zhang et al. [33] in 2018. Collectively, our findings contribute important evidence regarding the waning pattern of VZV antibodies and support the optimization of vaccination strategies, particularly the administration of a second dose beginning at four years of age to sustain protective immunity. Nevertheless, because our study was cross-sectional, these findings on the waning trend need to be further validated by consistent cohort studies that monitor antibody levels in vaccinated children over time.

Our study has several limitations. Firstly, this was a cross-sectional study, which cannot follow the individual evolution of antibodies, nor can it demonstrate the phenomenon of waning immunity. Secondly, the ELISA method may have underestimated the antibody positivity rate due to its limited sensitivity in detecting antibodies. Thirdly, the history of varicella infection was primarily based on self-reporting by participants, which may introduce recall bias. Antibodies induced by vaccines or natural infection cannot be distinguished, making it impossible to accurately estimate varicella infection in these populations. Finally, we only sampled serum from participants in Hangzhou and Quzhou, so geographical differences should be considered in future studies.

## 5. Conclusions

This observational population-based study assessed VZV seroepidemiology in healthy individuals aged 1 to 69 years in Zhejiang, China. Most children aged 1–6 years were susceptible to VZV, with persistently low antibody titers, whereas anti-VZV IgG seropositivity increased steadily with age and was nearly universal among adults. The coverage of VarV appears to be insufficient, and varicella is widely circulating in Zhejiang. These findings support the current two-dose recommendation for children and highlight the need to incorporate VarV into the national immunization program.

## Figures and Tables

**Table 1:** Analysis of seropositivity of varicella in general population.

Characteristics	Total (*N*)	Seropositivity		Univariate Analysis		Multivariate Analysis	
		*n*	% (95%CI)	χ^2^	*p*	OR (95%CI)	*p*
**Sex**				0.239	0.625		
male	381	275	72.18 (67.39–76.62)				
female	426	314	73.71 (69.26–77.83)				
**Age (years)**				223.857	<0.001		
1–6	100	41	41.00 (31.26–51.29)			1	
7–12	104	43	41.35 (31.77–51.42)			0.83 (0.47–1.48)	0.530
13–17	95	45	47.37 (37.03–57.88)			1.17 (0.65–2.10)	0.604
18–29	106	88	83.02 (74.50–89.61)			7.22 (3.33–15.69)	<0.001
30–39	103	88	85.44 (77.12–91.61)			8.55 (3.82–19.16)	<0.001
40–49	102	94	92.16 (85.13–96.55)			20.31 (8.03–51.37)	<0.001
50–59	100	96	96.00 (90.07–98.90)			42.16 (13.25–134.14)	<0.001
60–69	97	94	96.91 (91.23–99.36)			55.43 (15.28–201.06)	<0.001
**Residence**				1.603	0.206		
rural	512	366	71.48 (67.36–75.36)				
urban	295	223	75.59 (70.28–80.39)				
**History of varicella immunization**				128.049	<0.001		
no	584	488	83.56 (80.30–86.48)			1	
1 dose of vaccination	82	28	34.15 (24.07–45.45)			0.89 (0.46–1.73)	0.737
2 doses of vaccination	141	73	51.77 (43.21–60.26)			1.99 (1.11–3.55)	0.020
**History of varicella disease**				15.555	<0.001		
no	731	519	71.00 (67.56–74.27)			1	
yes	76	70	92.11 (83.60–97.05)			3.58 (1.43–8.94)	0.006
**Total**	807	589	73.08 (69.87–76.11)				

**Table 2:** Analysis of IgG antibody levels of varicella in general population.

Characteristics	Total (*N*)	GMC (mIU/mL, 95%CI)	Univariate Analysis		Multivariate Analysis	
			t/F	*p*	Β (95%CI)	*p*
**Sex**			0.603	0.547		
male	381	207.28 (180.08–238.59)				
female	426	195.49 (171.78–222.46)				
**Age (years)**			232.2	<0.001		
1–6	100	74.05 (57.09–96.05)			0	
7–12	104	82.82 (63.83–107.46)			−0.04 (−0.37–0.29)	0.800
13–17	95	102.91 (76.92–137.69)			0.27 (−0.06–0.61)	0.111
18–29	106	225.07 (173.35–292.21)			1.22 (0.82–1.62)	<0.001
30–39	103	300.64 (233.11–387.74)			1.50 (1.10–1.91)	<0.001
40–49	102	368.74 (304.29–446.85)			1.78 (1.38–2.18)	<0.001
50–59	100	441.45 (370.11–526.55)			1.97 (1.57–2.37)	<0.001
60–69	97	378.98 (324.64–442.42)			1.82 (1.42–2.23)	<0.001
**Residence**			1.206	0.228		
rural	512	216.58 (186.89–251.00)				
urban	295	192.49 (170.12–217.81)				
**History of varicella immunization**			72.17	<0.001		
no	584	269.92 (243.08–299.72)			0	
1 dose of vaccination	82	60.34 (45.65–79.75)			−0.09 (−0.45–0.28)	0.643
2 doses of vaccination	141	119.25 (96.88–146.78)			0.62 (0.29–0.95)	<0.001
**History of varicella disease**			−5.062	<0.001		
no	731	189.35 (171.09–209.56)			0	
yes	76	356.37 (284.11–447.01)			0.43 (0.14–0.73)	0.004
**Total**	807	200.97 (182.75–221.01)				

**Table 3:** Seropositivity and GMCs of anti-VZV IgG in VarV vaccination participants aged 1–14 years.

Characteristics	Total (*N*)	Seropositivity		χ^2^	*p*	GMC (mIU/mL, 95%CI)	t/F	*p*
		*n*	% (95%CI)					
**History of varicella immunization**				8.240	0.016		20.320	<0.001
no	53	16	30.19 (18.34–44.34)			49.96 (34.23–72.93)		
1 dose of vaccination	63	23	36.51 (24.73–49.60)			61.45 (45.36–83.26)		
2 doses of vaccination	116	60	51.72 (42.26–61.10)			120.55 (95.54–152.11)		
**History of varicella disease**				1.560	0.212		−2.725	0.068
no	227	95	41.85 (35.36–48.56)			79.57 (66.99–94.52)		
yes	5	4	80.00 (28.36–99.50)			238.85 (79.00–453.37)		
**Total**	232	99	42.67 (36.22–49.31)			82.10 (69.14–97.48)		

**Table 4:** VZV IgG antibody response across different post-vaccination intervals.

Varicella Vaccine	Years After Vaccination	Total (*N*)	Seropositivity (%, 95%CI)	*p*	GMC (mIU/mL, 95%CI)	*p*
1 dose	<1	13	61.54 (31.58–86.14)	0.312	94.81 (45.08–199.37)	0.151
	1-	14	35.71 (12.76–64.86)		70.82 (38.04–131.85)	
	2-	8	25.00 (3.19–65.09)		39.47 (16.22–96.09)	
	3-	3	33.33 (0.84–90.57)		86.23 (2.92–547.06)	
	4-	25	28.00 (12.07–49.39)		50.12 (29.49–85.18)	
2 doses	<1	32	71.88 (53.25–86.25)	0.044	195.01 (124.62–305.17)	0.007
	1-	15	60.00 (32.29–83.66)		121.32 (74.12–198.56)	
	2-	10	50.00 (18.71–81.29)		150.02 (49.96–450.52)	
	3-	23	34.78 (16.38–57.27)		87.35 (55.91–136.48)	
	4-	36	41.67 (25.51–59.24)		90.64 (56.74–144.81)	
overall	<1	45	68.89 (53.35–81.83)	0.008	158.33 (108.16–231.78)	0.003
	1-	29	48.28 (29.45–67.47)		93.56 (63.85–137.08)	
	2-	18	38.89 (17.30–64.25)		82.88 (39.78–172.81)	
	3-	26	34.62 (17.21–55.67)		87.22 (57.26–132.86)	
	4-	61	36.07 (24.16–49.37)		71.10 (50.11–100.87)	

## Data Availability

The data presented in this study are available on request from the corresponding author.

## Abbreviations

The following abbreviations are used in this manuscript:

VZV	Varicella zoster virus
VarV	Varicella attenuated live vaccine
WHO	World Health Organization
GMC	Geometric mean concentrations
IgG	Immunoglobulin G
ELISA	Enzyme-linked immunosorbent assay
